# Nanostructural engineering presents an opportunity for next-generation multi-dimensional camouflage

**DOI:** 10.1038/s41377-026-02224-6

**Published:** 2026-03-02

**Authors:** Bing Wei, Junjie Du

**Affiliations:** State Key Laboratory of Precision Spectroscopy, School of Physics, East China Normal University, Shanghai, China

**Keywords:** Imaging and sensing, Applied optics

## Abstract

A multidimensional camouflage approach is proposed to effectively counter the combined hyperspectral, thermal infrared, and polarization detection. Its innovative integration of surface roughening and silver nanomesh offers enhanced optical and thermal management, with potential applications in military stealth, environmental monitoring, and advanced defense technologies.

## Abstract (web summary)

A tri-layer camouflage coating integrates surface roughening and silver nanomesh to counter hyperspectral, thermal, and polarization detection, enhancing stealth capabilities for military and environmental applications.

## Graphical abstract

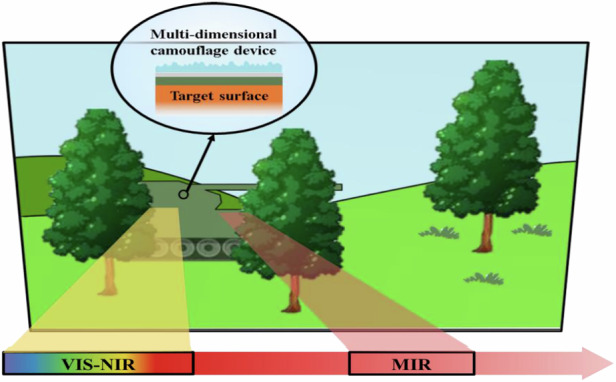

Camouflage is the technique of concealing objects by blending them into their environment or by mimicking other entities. In nature, this manifests through adaptive strategies: for example, chameleons alter their skin color, and leaf insects replicate the appearance of foliage to evade predators. In military contexts, camouflage is essential for concealing personnel, equipment, and installations, significantly reducing the risk of detection through visual, infrared, and radar surveillance. This ability directly enhances battlefield survivability and operational advantage, underscoring the crucial role camouflage plays in modern defense systems.

The effectiveness of camouflage, however, is fundamentally challenged by the wide spectrum of electromagnetic detection. Modern sensing spans a broad spectral range—from visible and near-infrared (NIR) to mid-wave/long-wave infrared (MWIR/LWIR) and radar. Consequently, to truly ‘blend in’ or ‘mimic’ a background, an object must align not only in color and shape but also in surface temperature and thermal radiative properties. This multidimensional challenge integrates optics, thermotics, and electromagnetics, requiring not just material development and application but also a complex design science.

Traditional detection methods primarily rely on measuring the intensity of electromagnetic signals across various spectral bands, which gives rise to techniques such as visible imaging, infrared imaging, thermal imaging, and radar. The coordinated use of these intensity-based methods forms the backbone of multidimensional surveillance systems, creating a formidable obstacle for traditional camouflage approaches. Overcoming this integrated detection process is the first step in the evolution of stealth technology, setting the stage for more advanced countermeasures. The diversity of electromagnetic signals—spanning intensity, spectral composition, polarization state, and spatial coherence—means that achieving true stealth requires engaging with a much richer and more complex set of information. This highlights the need for advanced, integrated countermeasures as we move toward the next frontier of camouflage technology.

The emergence of two new detection technologies is propelling counter-camouflage strategies into a new era of multi-physical-feature synergistic sensing. One of the latest advancements involves assigning “colors” to objects in the NIR spectrum by analyzing their unique spectral signatures^1–5^. Just as an object’s visible color is determined by its specific reflection and absorption patterns, it also has a distinct spectral fingerprint in the NIR band. For instance, plant leaves exhibit a sharp increase at the red edge, a plateau in the NIR range, and a gradual decline between 1.3 μm and 2.5 μm. Comparing these spectral signatures across the visible and NIR ranges allows for the detection of discrepancies, a process known as hyperspectral detection. Another key advancement is the use of polarization as a detection method. Polarization, an inherent property of transverse waves, provides an independent channel of information that is highly sensitive to an object’s surface structure, geometry, and material composition. By complementing traditional intensity-based and spectral imaging techniques, polarization sensing significantly enhances the accuracy of target classification and recognition^6–9^.

New detection methods, particularly polarization and hyperspectral sensing, are emerging as critical components in the detection landscape, posing significant challenges to conventional camouflage techniques. In response, Rui Qin et al. recently developed a multidimensional camouflage coating designed to counter hyperspectral, thermal infrared, and polarization detection simultaneously in their newest work published in *Light: Science & Applications*^10^. This innovative coating, applied to ground targets in vegetated environments, achieves a remarkable 96.9% spectral similarity with the surrounding vegetation. As a result, all three classification algorithms used for hyperspectral analysis failed to distinguish the coated target from the background. In contrast, a commercially available camouflage fabric was effective under only one of these algorithms. The coating’s effectiveness in polarization camouflage is equally impressive. Natural backgrounds typically exhibit low degrees of linear polarization (DoLP), while smooth man-made surfaces tend to show high DoLP at large observation angles, increasing their detectability. The new coating, however, maintains a very low DoLP—below 1.5% even at angles exceeding 70 degrees—effectively evading detection by polarization cameras.

The coating design utilizes a tri-layer architecture to achieve multidimensional camouflage, as shown in Fig. 1a, where each layer is carefully engineered to address a specific detection modality while maintaining compatibility with the others. The bottom layer consists of a WPU/MgCl₂·6H₂O matrix doped with Cr₂O₃, engineered to mimic the spectral ‘color’ of vegetation across the visible and NIR ranges (WPU refers to waterborne polyurethane), as displayed in Fig. 1b. The middle layer is a silver nanomesh, which effectively suppresses LWIR thermal radiation. The top layer is a highly transparent polyethylene film with a microscopically roughened surface, designed specifically to reduce the DoLP of emitted thermal radiation. The effectiveness of the upper two layers is evident: surface roughening efficiently depolarizes the thermal radiation (Fig. 1c), and the use of silver nanomesh, instead of conventional indium tin oxide (ITO), enhances visible-to-NIR transmittance while maintaining LWIR suppression. This carefully engineered nanomesh spacing strikes a reasonable balance between optical and thermal management. The high transparency of the top and middle layers ensures that the spectral signature of the Cr₂O₃ composite layer dominates, allowing for effective mimicry of the surrounding plant background’s broad-spectrum color.

**Fig. 1:**
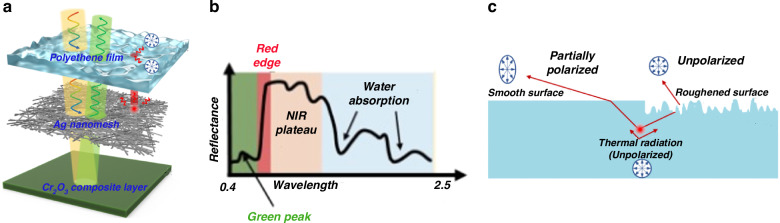
Schematic and working principles of the multidimensional camouflage scheme. **a** Tri-layer architecture of the designed coating, consisting of a polyethylene film (top), a silver (Ag) nanomesh (middle), and a Cr₂O₃ composite layer (bottom). **b** Effective imitation of key plant spectral characteristics (e.g., the red-edge rise) by the Cr₂O₃ composite layer. **c** Contrast in the polarization state of thermally emitted radiation between a rough surface and a smooth surface

Exploring strategies to counter emerging polarization and hyperspectral detection, while also balancing thermal radiation, visible, and NIR imaging, will be a key focus of future camouflage research. The foundational design principles demonstrated in this study—such as microroughened surfaces for depolarization and engineered metallic nanomeshes for thermal and optical management—provide a promising framework for future camouflage systems. Further refinement of these approaches will likely lead to their widespread adoption in next-generation multidimensional camouflage technologies. Looking ahead, two key areas of development will shape the future of camouflage: one is improving thermal performance, specifically by reducing thermal emissivity to maintain effectiveness in environments with significant temperature differences between the target and its surroundings. The other is enabling dynamic, adaptive camouflage. The integrated metallic grid, which acts as a transparent electrode, offers a potential pathway for seamless integration with tunable technologies like electrochromic or thermochromic materials, allowing for active control over spectral and thermal signatures. Expanding beyond vegetative environments, future camouflage technologies must also address the challenge of simulating diverse natural landscapes, such as desert terrains. This will require the development of advanced materials capable of precisely replicating the spectral signatures of sand, soil, and other natural elements. Success in this area will depend on the ability to reproduce subtle mineral absorption features through advanced chemical compositions and microstructural designs, pushing the boundaries of material science for camouflage.
